# Mixed POT-BM Approach for Modeling Unhealthy Air Pollution Events

**DOI:** 10.3390/ijerph18136754

**Published:** 2021-06-23

**Authors:** Nurulkamal Masseran, Muhammad Aslam Mohd Safari

**Affiliations:** 1 Department of Mathematical Sciences, Faculty of Science and Technology, Universiti Kebangsaan Malaysia, UKM, Bangi 43600, Selangor, Malaysia; 2 Department of Mathematics, Faculty of Science, Universiti Putra Malaysia, UPM, Serdang 43400, Selangor, Malaysia; aslam.safari@upm.edu.my

**Keywords:** air pollution modeling, environmetrics, pollution risk assessment

## Abstract

This article proposes a novel data selection technique called the mixed peak-over-threshold–block-maxima (POT-BM) approach for modeling unhealthy air pollution events. The POT technique is employed to obtain a group of blocks containing data points satisfying extreme-event criteria that are greater than a particular threshold *u*. The selected groups are defined as POT blocks. In parallel with that, a declustering technique is used to overcome the problem of dependency behaviors that occurs among adjacent POT blocks. Finally, the BM concept is integrated to determine the maximum data points for each POT block. Results show that the extreme data points determined by the mixed POT-BM approach satisfy the independent properties of extreme events, with satisfactory fitted model precision results. Overall, this study concludes that the mixed POT-BM approach provides a balanced tradeoff between bias and variance in the statistical modeling of extreme-value events. A case study was conducted by modeling an extreme event based on unhealthy air pollution events with a threshold u > 100 in Klang, Malaysia.

## 1. Introduction

In the practical application of statistical modeling, the phenomena under investigation generally involve several rare events occurring with very high or very low values, relatively corresponding to numerous data points. The existence of such rare events can affect data distribution characteristics, such as in the form of high skewness and kurtosis, which correspond to long- and heavy-tail behaviors. In environmental phenomena, the occurrence of extreme events can be observed in various fields, such as extreme precipitation and flooding [1], extreme wind speeds [2], droughts [3], extreme temperatures [4], natural hazards [5], and air pollution events [6,7].

The presence of rare and extreme events leads to a heavy-tail property that makes commonly used normal/Gaussian models unsuitable for describing the data distribution [8,9]. Thus, to address this issue, two statistical extreme-value models for extreme events have been developed, namely the generalized extreme-value (GEV) and generalized Pareto distribution (GPD) models [10,11]. Both models have been found to be useful tools for providing good insights into the behaviors of extreme air pollution events [12,13,14,15,16,17,18,19,20,21]. For example, Al-Dhurafi et al. [12] employed the GPD model to represent probability distributions in an unhealthy air pollution index for three highly polluted urban areas in Peninsular Malaysia. Martins et al. [13] employed the GEV and GPD models to investigate the behavior of air pollutants in two large urban regions (Metropolitan Area of São Paulo and Metropolitan Area of Rio de Janeiro) in South America. Masseran et al. [18] used the GPD model as a tool to investigate the risk of occurrence of unhealthy air pollution events for eight urban areas in Peninsular Malaysia. Reyes et al. [19] provided an estimation of the high ozone levels trend for seven of the meteorological stations located in the Metropolitan Area of Mexico city using the quantile function determined from a fitted GEV model. Battista et al. [21] evaluated the behavior of the air pollution level in the city of Rome (Italy) using the GEV model. Overall, all of these studies found that the results obtained with extreme-value models provided valuable information to use as a basis of managing the risk of air pollution events.

Conceptually, the GEV model is derived from the block-maxima (BM) approach, which generally uses a data selection method based on annual maximum extreme events, and this implies that only one extreme event per year can be used for GEV modeling [22,23,24]. However, this process can lead to a loss of the information contained in other large-sample values [12,25]. In reality, hazardous phenomena that occur within a year may not only be those that exert a maximum effect but also those that are the second or third most serious, which may lead to extreme events in different time periods within the year [26]. The BM approach fails to represent actual phenomena involving the occurrence of multiple extreme events within a year [27]. Therefore, a single maximum point per year definitely reduces the quality of the data representing events of interest, which could significantly contribute to the outcome bias of the extreme value analysis [28]. Although the BM size can be adjusted to include additional extreme data points, no clear rules exist to determine the optimal block size for practical applications.

GPD modeling based on the peak-over-threshold (POT) approach is conducted to determine extreme events as data points greater than a particular threshold level [29,30]. This approach can enable flexible selection of extreme data points, allowing the inclusion of a wide range of extreme events in the analysis [31,32,33]. A high number of selected data points in the extreme-value modeling process provides better precision in terms of the inference and parameter estimation of the statistical model, as well as its quantile estimates [34,35]. However, extreme data points determined with the POT approach tend to lead to the problem of dependency behaviors, which implies that there is a bias in the statistical modeling process. Although the threshold can be adjusted to overcome the dependency behavior problem, the process of determining an optimal threshold can provide only a subjective solution. This subjective threshold selection may not be able to provide an interpretable meaning for the problem under investigation [12,18]. For a problem related to an unhealthy air pollution event (air pollution index (API) > 100), a fixed threshold *u* = 100 can provide a meaningful analysis [36].

In summary, the BM and POT approaches have their own advantages and limitations. Therefore, this article proposes an alternative approach by combining the BM and POT approaches to provide improved tradeoff results in terms of the bias and variance of the fitted model, as seen in particular for a practical application in air pollution modeling.

## 2. Study Area and API Data

**Figure 1:**
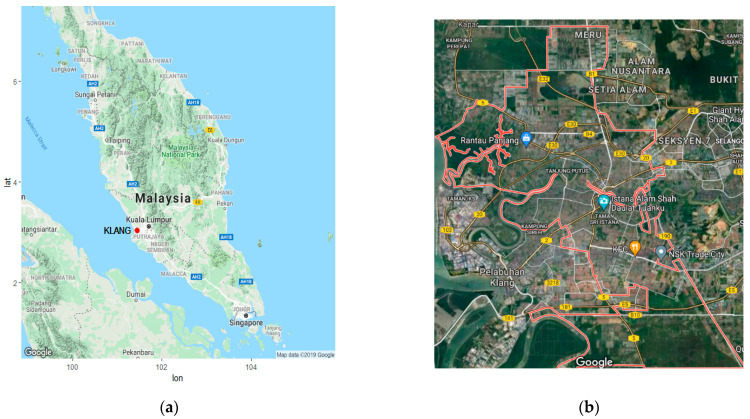
(**a**) Map of Peninsular Malaysia (Klang is identified by the red dot); (**b**) map of Klang.

Klang, which is located in Peninsular Malaysia at a latitude of 101°26′44.023 E and a longitude of 3°2′41.701 N, is one of Malaysia’s largest cities, with a land area of approximately 573 km^2^. The population density in Klang is among the highest of any city in Malaysia, 1034 people per square kilometer [37]. Klang is also the center of important industrial and economic interests of the country. Moreover, Klang has been recognized as the 13th busiest trans-shipment port and 16th busiest container port in the world [38,39]. However, the rapid development of urban commercial and industrial areas in Klang in recent decades has elevated its risk of atmospheric pollution. Therefore, given the importance of the city’s industrial activities, monitoring and evaluating the behavior of extreme pollution events in Klang is crucial. Figure 1 presents a map of Peninsular Malaysia with the location of Klang [40].

The API in Malaysia is derived from the five main pollutants: suspended particulate matter less than ten microns in size (PM_10_), nitrogen dioxide (NO_2_), carbon monoxide (CO), ozone (O_3_), and sulfur dioxide (SO_2_). Afroz et al. [41] reported that there are three major sources that influence the concentration levels for the CO, SO_2_, NO_2_, O_3_, and PM_10_ pollutant variables in Malaysia. They categorized thee sources as mobile sources, stationary sources, and open burning sources, which in turn can be decomposed into various factors, including industrial and construction activities, transportation exhaust emission, soil dust, open burning activity, haze events, and so on [42,43,44,45,46]. The observed data for CO, SO_2_, NO_2_, and O_3_ are quantified in terms of parts per million (ppm) units for the mass of a contaminant. The observed PM_10_ data are quantified in terms of micrograms per cubic meter (μg/m^3^). Thus, to measure the API indices, these pollutant variables need to be standardized to derive individual indices. Based on the observed data, the calculation of the sub-API indices can be undertaken using the mathematical formulas provided by the Department of Environment Malaysia [47]. The sub-API value for the CO pollutant can be computed using the following equation:

(1) $$Idx\left(CO\right)=\{\begin{array}{cc} \;CO\times 11.11111, & if\;\;CO<9\;\mathrm{ppm},\\ \;100+\left\{\left[CO-9\right]\times 16.66667\right\}, & if\;9\;\leq CO<15\;\mathrm{ppm},\\ 200+\left\{\left[CO-15\right]\times 6.66667\right\}, & if\;15\;\leq CO<30\;\mathrm{ppm},\\ \;300+\left\{\left[CO-30\right]\times 10\right\}, & if\;\;\;\;CO\geq 30\;\mathrm{ppm}. \end{array}$$

The sub-API value for the O_3_ pollutant can be computed using the following equation:

(2) $$Idx\left(O_{3}\right)=\{\begin{array}{cc} \;O_{3}\times 1000, & if\;\;O_{3}<0.2\;\mathrm{ppm},\\ 200+\left\{\left[O_{3}-0.2\right]\times 500\right\}, & if\;0.2\;\leq CO<0.4\;\mathrm{ppm},\\ \;300+\left\{\left[O_{3}-0.4\right]\times 1000\right\}, & if\;\;\;O_{3}\geq 0.4\;\mathrm{ppm}.\;\; \end{array}$$

The sub-API value for the NO_2_ pollutant can be computed using the following equation:

(3) $$Idx\left(NO_{2}\right)=\{\begin{array}{cc} \;\;NO_{2}\times 588.23529, & if\;\;NO_{2}<0.17\;\mathrm{ppm},\\ 100+\left\{\left[NO_{2}-0.17\right]\times 232.56\right\}, & if\;0.17\;\leq NO_{2}<0.6\;\mathrm{ppm},\\ \;200+\left\{\left[NO_{2}-0.6\right]\times 166.667\right\}, & if\;0.6\;\leq NO_{2}<1.2\;\mathrm{ppm},\\ \;\;300+\left\{\left[NO_{2}-1.2\right]\times 250\right\}, & if\;\;\;\;NO_{2}\geq 1.2\;\mathrm{ppm}. \end{array}$$

The sub-API value for the SO_2_ pollutant can be computed using the following equation:

(4) $$Idx\left(SO_{2}\right)=\{\begin{array}{cc} \;\;SO_{2}\times 2500, & if\;\;SO_{2}<0.04\;\mathrm{ppm},\;\\ 100+\left\{\left[SO_{2}-0.04\right]\times 384.61\right\}, & if\;0.04\;\leq SO_{2}<0.3\;\mathrm{ppm},\\ \;200+\left\{\left[SO_{2}-0.3\right]\times 333.333\right\}, & if\;0.3\;\leq SO_{2}<0.6\;\mathrm{ppm},\\ \;\;300+\left\{\left[SO_{2}-0.6\right]\times 500\right\}, & if\;\;\;\;SO_{2}\geq 0.6\;\mathrm{ppm}. \end{array}$$

The sub-API value for the PM_10_ pollutant can be computed using the following equation:

(5) $$Idx\left(PM_{10}\right)=\{\begin{array}{cc} \;\;\;PM_{10}, & if\;\;\;PM_{10}<50\;{\mathrm{ug}/m}^{3},\\ \;50+\left\{\left[PM_{10}-50\right]\times 0.5\right\}, & if\;\;50\;\leq PM_{10}<350\;{\mathrm{ug}/m}^{3},\\ 200+\left\{\left[PM_{10}-350\right]\times 1.4286\right\}, & if\;\;\;350\;\leq PM_{10}<420\;{\mathrm{ug}/m}^{3},\\ \;300+\left\{\left[PM_{10}-420\right]\times 1.25\right\}, & if\;\;420\;\leq PM_{10}<500\;{\mathrm{ug}/m}^{3},\\ \;\;400+\left[PM_{10}-500\right], & if\;\;\;\;\;PM_{10}\geq 500\;{\mathrm{ug}/m}^{3}.\; \end{array}$$

**Figure 2:**
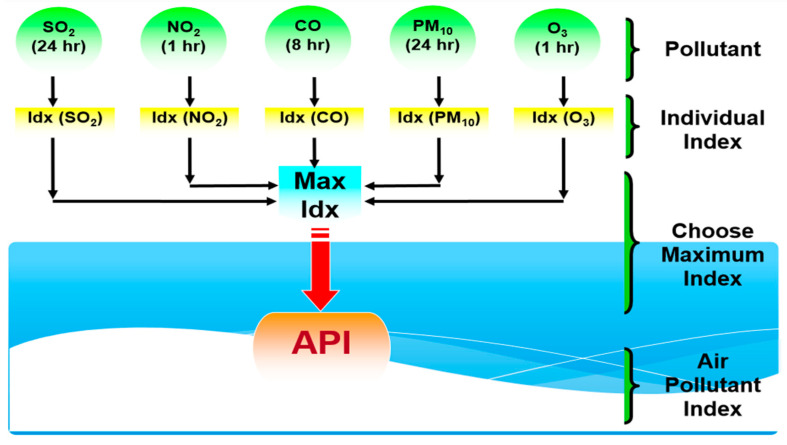
The process of determining the API value.

Based on these individual indices, the API value at a particular time can be determined based on the highest value among these sub-indices [48,49]. Figure 2 shows a schematic illustration of the process of determining the API value. Table 1 describes the air quality statuses corresponding to API values at particular time [47].

The higher the API value at a particular time, the greater the threat of the occurrence of extreme pollution events. Such scenarios disrupt the economic activities of the country, lead to a high risk health problems among populations, and negatively affect environmental ecosystems. Thus, it is important to investigate the behavior of air pollution events in order to gain an understanding of these issues. This study used hourly API data from Klang for the period from 1 January 1997 to 31 August 2020 as a case study. Details of the raw API data and how to request access are available on the Department of Environment Malaysia website [50]. This study was interested in the occurrence of extreme air pollution events determined by unhealthy API indices.

## 3. Extreme-Value Modeling

### 3.1. BM Approach

In the BM approach, the extreme behavior of an air pollution event can be evaluated based on a maximum recorded API value determined from a particular period, which is defined as a block. If we let $X_{1},X_{2},\ldots ,X_{n}$ be a random variable representing API data following a particular density function *F*, then the probability behavior of the maximum API value from a particular block can be written as $Y=\max\left(X_{1},X_{2},\ldots ,X_{n}\right)$. The density function of the random variable Y is determined from the following equation:

(6) $$P\left(Y\leq y\right)=P\left(X_{1}\leq x,X_{2}\leq x,\ldots ,X_{n}\leq y\right)=F^{n}\left(y\right).$$

The density of $F^{n}$ can be used as an accurate approximation model for probability distribution of the variable $Y=\max\left(X_{1},X_{2},\ldots ,X_{n}\right)$ despite the independent and identical conditions of the original random variable $X_{1},X_{2},\ldots ,X_{n}$ not being satisfied. This flexibility makes conducting extreme-value analysis based on the BM approach for various types of problems, including those involving complex phenomena, plausible [51].

In a practical application involving a dataset, the actual density function *F* representing the distribution of the phenomenon under investigation is unknown. This concept implies that the distribution of *F* must first be estimated from the observed data before it can be used in Equation (6). However, small discrepancies in the *F* determination will lead to substantial discrepancies in $F^{n}$. In parallel, subsequent analyses using the $F^{n}$ density function will contribute to large errors, consequently leading to incorrect results [52]. To overcome this problem, the density function *F* can be assumed to be unknown, whereas the determination of the $F^{n}$ density function can be approximated to a particular limiting distribution form as n→∞. Mathematically, this limiting distribution *G(y)* is valid if a sequence of constants $\left\{a_{n}>0\right\}$ and $\left\{c_{n}\right\}$ exists, such that:

(7) $$P\left\{\frac{Y-c_{n}}{a_{n}}\leq y\right\}\to G\left(y\right),\;\;as\;n\to \infty ,$$

where *G(y)* is also a nondegenerate function [53]. According to the literature, the limiting distribution in Equation (7) provides the form of the GEV distribution as the following equation:

(8) G(y)={exp{−[1−κ(y−ξα)]1κ}, for κ≠0,exp[−exp{−(y−ξα)}], for κ=0,

where the location, scale, and shape parameters are denoted by the notations ξ, α, and κ, respectively. These parameters must be estimated to provide a practical distribution function that can be used to represent the data distribution. However, owing to the problem of small sample size deriving from the BM approach, estimation using the likelihood-based approach, such as maximum likelihood estimation, will not generate satisfactory results. As described by Hosking et al. [54], the L-moment method is an effective alternative approach to overcome this issue. The L-moment estimators of the GEV model are given as follows:

(9) $$\hat{\xi }={\hat{\lambda }}_{1}-\frac{\hat{\alpha }}{\hat{\kappa }}\left\{1-\Gamma \left(1+\hat{\kappa }\right)\right\},$$

(10) $$\hat{\alpha }=\frac{{\hat{\lambda }}_{2}\hat{\kappa }}{\left(1-2^{-\hat{\kappa }}\right)\Gamma \left(1+\hat{\kappa }\right)},$$

(11) $$\hat{\kappa }=7.859c+2.9554c^{2},$$

where $c=\frac{2}{\left(3+{\hat{\tau }}_{3}\right)}-\frac{\log\left(2\right)}{\log\left(3\right)}$. In conjunction with Equations (9)–(11), the L-moment estimators of the terms ${\hat{\lambda }}_{1},\;{\hat{\lambda }}_{2},\;{\hat{\lambda }}_{3}$ and ${\hat{\tau }}_{3}={\hat{\lambda }}_{3}/{\hat{\lambda }}_{2}$ must be estimated from the probability weighted moments determined from the following equation:

(12) $$\beta_{r}=\xi +\frac{\alpha \left[1-{\left(r+1\right)}^{-\kappa }\Gamma \left(1+\kappa \right)\right]}{\kappa \left(r+1\right)},$$

where $\lambda_{1}=\beta_{0}$, $\lambda_{2}=2\beta_{1}-\beta_{0}$, and $\lambda_{3}=6\beta_{2}-6\beta_{1}+\beta_{0}$. The unbiased estimator of $\beta_{r}$ is determined as follows:

(13) $$b_{r}=\sum_{i=1}^{n}\left[\frac{\left(i-1\right)\left(i-2\right)\left(i-3\right)\ldots \left(i-r\right)}{n\left(n-1\right)\left(n-1\right)\ldots \left(n-r\right)}y_{\left(i\right)}\right],\;for=0,\;1,\;2,\ldots ,$$

where $y_{\left(i\right)}$ is the order statistic determined from the sample data (for additional details on the L-moment estimators of the GEV model, refer to [55,56,57]). The quantile function of the GEV model can be obtained by inverting its cumulative density function (CDF) [58], which is given as follow:

(14) $$G^{-1}\left(p,\xi ,\alpha ,\kappa \right)=\{\begin{array}{c} \xi +\frac{\alpha }{\kappa }\left[1-{\left(-\ln\left(p\right)\right)}^{\kappa }\right],\;for\;\kappa \neq 0,\\ \xi -\alpha \ln\left(-\ln\left(p\right)\right),\;\;\;for\;\kappa =0. \end{array}$$

The quantile function knowledge in Equation (14) is crucial for determining the return period related to an extreme pollution event. The return period, referring to the probability of the time (T) block extreme event being exceeded, is 1T in every time block period. Thus, for any particular return period $x_{T}$, the critical value of the maximum API level can be determined using the following equation:

(15) $$P\left(Y>y_{T}\right)=G\left(y_{T}\right)=\frac{1}{T}.$$

In parallel, the maximum API level corresponds to a particular period *T*, which can be computed as follows:

(16) $$y_{T}=G^{-1}\left(\frac{T-1}{T}\right).$$

### 3.2. POT Approach

This approach uses a particular threshold value to isolate data points considered extreme from the rest of the dataset before determining a statistical model to describe an extreme phenomenon. To relate this approach to the analysis of an unhealthy air pollution event, based on Table 1, an API value exceeding the threshold of 100 is determined as the unhealthy air pollution event. Thus, a fixed threshold value indicated by *u* = 100 has a significant meaning in air pollution studies. Let $X_{1},X_{2},\ldots ,X_{n}$ represent a random variable in the API data following an unknown density function *F*. Mathematically, an unhealthy air pollution event phenomenon can be represented by a conditional event corresponding to a threshold greater than *u*. The conditional exceedance distribution function $F^{\left[u\right]}$ is determined as follows:

(17) $$\begin{array}{l} F^{\left[u\right]}\left(x\right)=\mathrm{Pr}\left(X\leq x|X>u\right)\\ \;\;\;=\frac{\mathrm{Pr}\left\{X\leq x,X>u\right\}}{\mathrm{Pr}\left\{X>u\right\}}\\ \;\;\;=\frac{F\left(x\right)-F\left(u\right)}{1-F\left(u\right)},\;\;x\geq u. \end{array}$$

This conditional exceedance distribution function has a left endpoint of $F^{\left[u\right]}$; that is, $\alpha \left(F^{\left[u\right]}\right)=\inf\left(y:F^{\left[u\right]}\left(y\right)>0\right)$ equal to 0. However, if we let Y=X−u represent a random variable in the data above the threshold *u*, then the empirical cumulative density function ${\hat{F}}_{k}\left(y;.\right)$ is given by the following equation:

(18) $${\hat{F}}_{k}\left(y;.\right)={\left({\hat{F}}_{k}\left(y;.\right)\right)}^{\left[u\right]}.$$

Equations (17) and (18) provide the same information [51]. To determine the parametric form of this density function, the limiting distribution of the normalized values exceeding the threshold must be derived. According to the literature, as the threshold approaches the endpoint of the variable, the limiting distribution of the cumulative density in Equation (18) will follow a GPD [11,59], which can be given as follows:

(19) F(x)=P(X≤x|X>u)={1−(1+ξ(x−uσ))−1ξ,if ξ≠0,1−e−(x−uσ),if ξ=0.

Meanwhile, in terms of the random variable Y=X−u, we obtain:

(20) G(y)=P(Y≤y)={1−(1+ξyσ)−1ξ,if ξ≠0,1−e−yσ,if ξ=0,

where $1+\frac{\xi y}{\sigma }>0$. The threshold, shape, and scale parameters are represented by u, ξ, and σ, respectively [60,61]. The tail behavior influenced by the existence of extreme data can be described by the shape parameter ξ. If ξ=0, then the right upper-tail distribution of the data indicates the properties of a medium-sized tail, which implies that the GPD can be approximated to an exponential distribution. On the one hand, if ξ>0, then it indicates the properties of a short-tailed distribution, which leads the GPD approximation to a Pareto type II model. On the other hand, if ξ<0, then it indicates the existence of long-tailed behavior that can approximate the GPD to an ordinary Pareto distribution. Moreover, the mean of the data exceeding the threshold *u* can be described by the scale parameter σ [25].

To determine the parameter estimates of the GPD model, a method based on its likelihood function can be used, as large data points can commonly be obtained for the POT approach. For the values of $y_{1},y_{2},\ldots ,y_{k}$, where *k* is the total number of data points exceeding the threshold *u*, the log-likelihood function of the GPD is given as follows:

(21) log(L)=−klogσ−(1+1ξ)∑i=1klog(1+ξyiσ).

However, no analytical solution can be obtained from Equation (21). Thus, a numerical procedure must be utilized to obtain a final solution for the parameter estimation.

Next, similar to the GEV model, the concept of the return period can be employed in the GPD model. A return period is interpreted as the average period between extreme events exceeding a threshold *u* within a particular period of time [27], which is derived based on knowledge of its distribution function. The return period of the GPD model is given as the following equation [53]:

(22) $$P\left(Y>y|Y>u\right)={\left[1+\xi \left(\frac{y-u}{\sigma }\right)\right]}^{-\frac{1}{\xi }}.$$

If we let $\zeta_{u}=P\left(X>u\right)=\frac{k}{n}$, where *k* is the number of data points $x_{i}$ exceeding the threshold *u*, then the return period presented in Equation (22) can be simplified as:

(23) $$P\left(Y>y\right)=\zeta_{u}{\left[1+\xi \left(\frac{y-u}{\sigma }\right)\right]}^{-\frac{1}{\xi }},$$

which implies that the API level exceeded once every *m* series of observations on average can be determined using the following equation:

(24) $$\frac{1}{m}=\zeta_{u}{\left[1+\xi \left(\frac{y_{u}-u}{\sigma }\right)\right]}^{-\frac{1}{\xi }}.$$

Specifically, for an unhealthy air pollution event indicated by threshold *u* = 100, Equation (24) can be simplified as

(25) $$y_{u}=u+\frac{\sigma }{\xi }\left[{\left(m\zeta_{u}\right)}^{\xi }-1\right].$$

In addition, by manipulating the return period formula, information on the expected return level corresponding to a particular return period can be obtained [62] as follows:

(26) $$u=G^{-1}\left(1-\frac{1}{P_{R}\left(u\right)}\right).$$

where $G^{-1}$ is the quantile function of the GPD model.

### 3.3. Mixed POT-BM Approach

The relationship between the GEV model (BM approach) and GPD model (POT approach) can be described using asymptotic model characterization [63]. As described above, in a sequence of independent random variables $X_{1},X_{2},\ldots ,X_{n}$ with a common density function *F*, the maximum value of data in a particular block is determined as $Y=\max\left(X_{1},X_{2},\ldots ,X_{n}\right)$. In a large *n*, the asymptotic density function of *Y* is P(Y≤y)≈G(y), where G(y) is the GEV model presented in Equation (8). In parallel, for a particular adequately large threshold *u*, the density function of the random variable X−u conditional on X>u can be approximated to the density function of the GPD model presented in Equation (20) [53]. In a mixed POT-BM approach, for a series of random variables $X_{1},X_{2},\ldots ,X_{n}$, the POT data are determined first by grouping them into a block containing data points satisfying the criteria of X>u. However, some extreme events may occur in sequence over time. For example, an extreme air pollution event will likely be followed by another extreme air pollution event for several consecutive hours or days. Thus, this scenario violates the independence assumption for the selected POT data points. To overcome this problem, a declustering technique can be used to filter dependent consecutive extreme values exceeding the threshold *u*. Declustering is performed by selecting only the POT data blocks with a minimum separation r from one another. According to Karim et al. [64], *r* = 240 h is sufficient as the minimum number of separation hours between two extreme events.

After the declustering of the POT blocks has been determined, the BM concept is applied to select the maximum value of the data points in each POT block. This step implies that the extreme data points selected by the mixed POT-BM approach can satisfy or at least satisfactorily approximate the properties of an independent variable. Thus, instead of making a pragmatic choice, such as a block size length of one year—as is common practice in the BM approach, leading to a small number of selected data points and to high variance in the statistical modeling—all data points exceeding the threshold *u* in the POT approach can be selected; however, this choice violates the asymptotic properties of the extreme-value model, thereby implying a high bias. The mixed POT-BM approach can be a reasonable alternative for providing a balanced tradeoff between bias and variance generated by the data selection in the extreme-value statistical modeling.

In a similar vein, based on the relationship between the GEV model (BM approach) and GPD model (POT approach) described above, the argument regarding this matter leads to an intuitive justification that, for a particular adequately large threshold *u*, several independent maximum block data governed by the GEV model can be obtained, which implies that the mixed POT-BM approach can achieve satisfactory behaviors similar to the BM approach. Thus, the density function corresponding to the mixed POT-BM approach can be approximated to the GEV model presented in Equation (8). However, the POT-BM approach can be a highly reliable method, as it can determine more extreme data points than the common BM approach. Large independent extreme data points determined using this approach increase GEV modeling precision, as a low estimation variance can be obtained. Moreover, the model bias problem can be avoided by integrating the declustering technique in the POT-BM approach.

By taking advantage of the large number of extreme data points generated by the POT-BM approach, the maximum likelihood can be used instead of the L-moments method for parameter estimation. According to Coles [53], the likelihood-based method is equipped with a convenient set of “off-the-shelf” inference properties and can quantify uncertainties during estimation. The log-likelihood function of the GEV model for κ≠0 can be written as

(27) $$\ell \left(\theta |y\right)=-n\log\left(\alpha \right)+\sum_{i=1}^{n}\left[\left(\frac{1}{\kappa }-1\right)\ln\left(z_{i}\right)-{\left(z_{i}\right)}^{\frac{1}{\kappa }}\right],$$

where $\theta ={\left[\xi ,\alpha ,\kappa \right]}^{\prime }$ and $z_{i}=\left[1-\left(\kappa /\alpha \right)\left(y_{i}-\xi \right)\right]$ [57,65]. However, the log-likelihood function of the GEV model presented in Equation (27) cannot provide a simple analytical solution for parameters ξ, α, and κ. Thus, parameters that can maximize the log-likelihood function can be determined via a numerical optimization technique, which is available in many programming language software, such as R [66].

## 4. Results and Discussion

**Figure 3:**
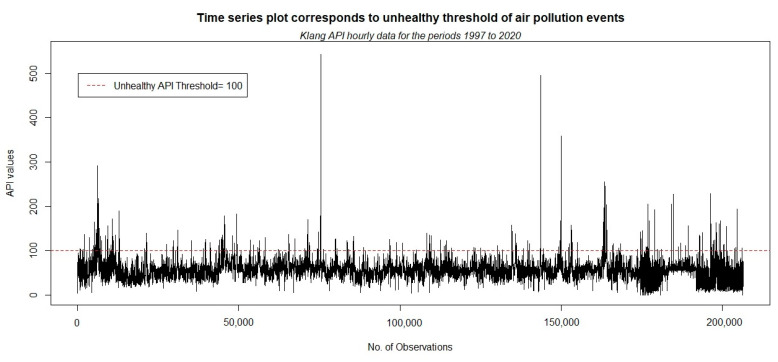
Time series plot corresponding to unhealthy air pollution event threshold.

Before a detailed analysis is conducted, deriving basic information on the descriptive statistics data would be worthwhile. Figure 3 illustrates the time series plot for the observed hourly API data in the Klang area for the period from 1 January 1997 to 31 August 2020, and Table 2 presents several descriptive measures of the data.

The information shows that most of the time, in healthy conditions, the API values in Klang fluctuate (below a threshold of 100) around the mean, which is 55.222. However, variance is relatively high (20.970). This high variability derives from unhealthy pollution events that occur repeatedly, as shown by the spike in the data points in the time series plot. The maximum API value recorded in the 24 year data is approximately 541, which indicates a hazardous air quality status (as described in Table 1). These anomalies of API values closely relate to the occurrence of haze events in Malaysia [67]. As reported by the Department of Environment Malaysia [68], haze episodes have recurrently occurred in the years 2005, 2012, 2014, and 2015. In particular, with regard to the API value of approximately 541, this scenario stems from a severe haze event that occurred on 11 August 2005. During that period, the government of Malaysia declared a state of emergency in the Klang area. This result implies that the unhealthy and extreme air pollution event risks in Klang should not be taken lightly. Meanwhile, the skewness and kurtosis measures indicate that the data are not normally distributed. This information can lead to the reasonable application of extreme-value modeling for the data.

**Figure 4:**
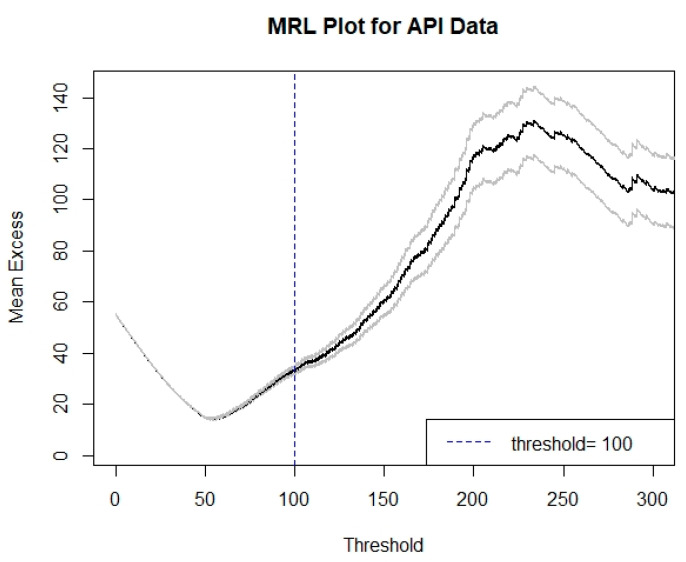
MRL plot corresponding to threshold *u* = 100.

Moreover, as an API greater than 100 indicates the occurrence of unhealthy air pollution events, a fixed threshold *u* = 100 was essential in this study as the basis for the extreme-value modeling and analysis. For the POT approach, the selected threshold can affect GPD modeling precision. Thus, as this study used a fixed threshold *u* = 100 to assess the suitability of a POT approach, a mean residual life (MRL) plot could be utilized. The MRL plot should indicate an approximate linear behavior in the threshold *u* [53]. Figure 4 shows the MRL plot for the API data. In the threshold *u* = 100, approximate linear behavior is satisfied. Thus, *u* = 100 is a valid threshold for extreme-value modeling using the GPD model. Table 3 provides the results of the parameter estimation for each fitted extreme-value model. The estimated parameters of the GEV fitted model based on the BM approach have the largest standard error compared with those of the other approaches, which implies less precise results. The parameters of the GPD fitted model based on the POT approach have the smallest standard error, which implies high precision. Moreover, the estimated parameters for the GEV approximation based on the POT-BM approach can provide a considerably reduced standard error compared with those of the GEV model based on the BM approach, and it is slightly higher than the standard error obtained by the GPD model based on the POT approach. This result indicates that the application of the GEV model with the BM approach was a less precise method to deal with the data of extreme air pollution events, while both the POT method and the GEV with POT-BM approach were found to produce better results with small standard errors. However, as described above, another important criterion that needs to be evaluated for these methods is their dependency behaviors in order to avoid the bias problem in extreme-value modeling.

**Figure 5:**
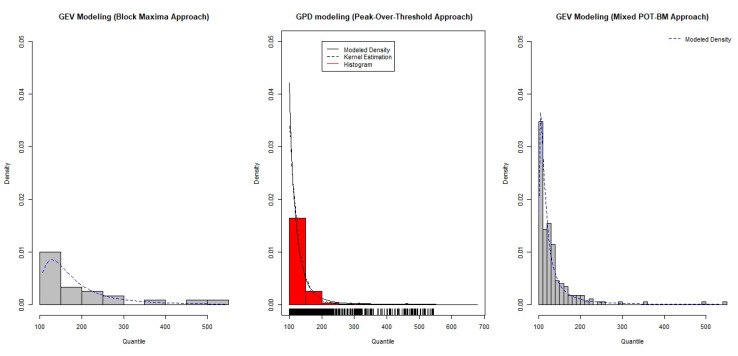
Comparison of fitted models.

**Figure 6:**
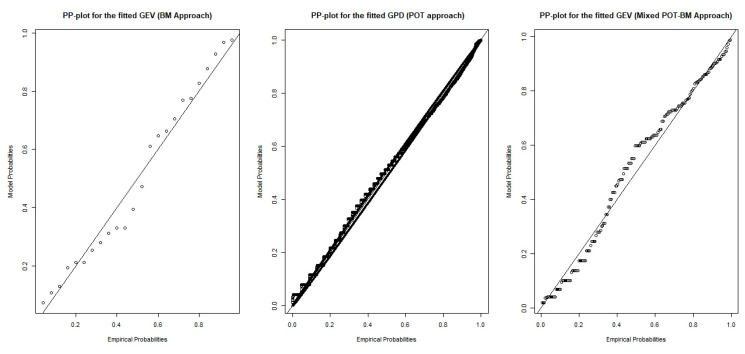
PP plot for each fitted model.

Based on the results of the parameter estimation, Figure 5 presents a comparison of the fitted extreme-value models that clearly shows the most precise model in terms of the parameter estimation standard error. According to Figure 5, the fitted density of the GEV model based on the BM approach provides relatively rough results, indicating that the less precise model could represent the occurrence of extreme phenomena in the dataset. Meanwhile, GPD modeling based on POT and GEV approximation based on the POT-BM approach could provide improved model fitting with high precision. The comparison of PP plots in Figure 6 accords with the results in Figure 5 and Table 3.

**Figure 7:**
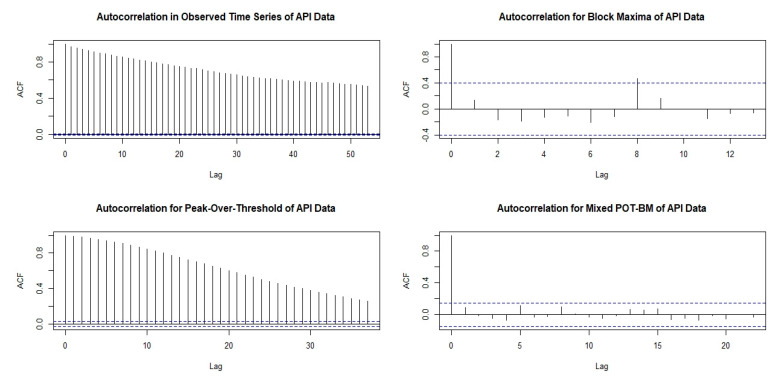
Autocorrelation function of data based on different extreme-value approaches.

Apart from the precision of the fitted models in terms of their standard error, representing variance in the model estimation, another important criterion is the independence assumption for the selected extreme data. Violation of the independence assumption leads to the bias problem in the fitted models. Figure 7 shows the autocorrelation function of the data based on the different extreme-value approaches. The original observed hourly API data (top left) have a very strong dependency on their previous time lag, which clearly violates the independence assumption. Thus, the use of the BM approach (top right) could eliminate the problem of dependency behaviors, whereas the use of the POT approach (bottom left) could slightly reduce dependency behaviors, which remained noticeably significant. Moreover, the use of the mixed POT-BM approach (bottom left) made it possible to solve the problem of dependency behaviors.

**Figure 8:**
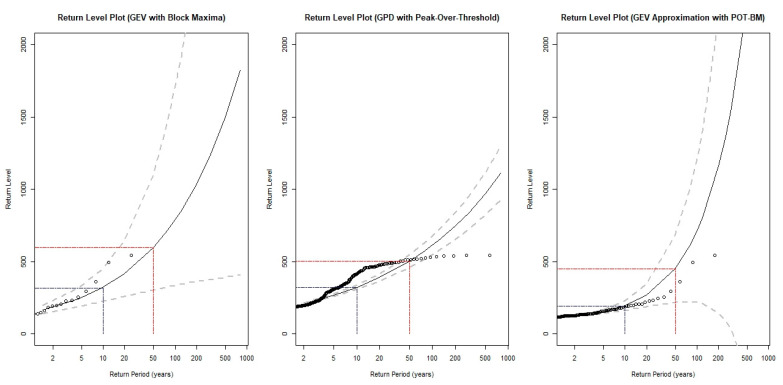
Comparison of return level plot of fitted models.

The highlighted result was obtained from the results presented in Table 3 and Figure 5, Figure 6 and Figure 7, which indicate that extreme-value modeling based on the BM approach is unbiased but less precise, with large estimation variance. However, though the POT approach tends to demonstrate high precision in extreme-value modeling, it suffers from bias due to the violation of the independence assumption. Meanwhile, the mixed POT-BM approach can provide an improved tradeoff between bias and variance. In addition, the mixed POT-BM approach can satisfy the unbiased properties of extreme-value modeling with much lower variance compared to the BM approach. In a similar vein, Figure 8 presents a comparison of the return level plot of each fitted extreme-value model. Based on Figure 8, the return level estimate determined by the GPD model based on the POT approach has a small confidence interval, but its estimation is clearly biased compared with the empirical data. For the return level estimate determined by the GEV model based on the BM approach, its precision with the empirical data is satisfactory. However, the return level plot derived from the GEV model with the POT-BM approach was found to produce a better result. The plot shows that the estimated return level curves within the range of the observed data are accurate with a small confidence interval, particularly in short return periods of time, such as 5 or 10 years. However, for a long return period, the confidence interval of the return level estimation is larger. This implies that the accuracy of the return level estimation decreases. Thus, to provide a better assessment, we suggest that the GEV (POT-BM) model needs to be re-fitted with a current set of data to obtain the most up-to-date short term (5 or 10 years of the future) evaluation of extreme pollution event over time.

This finding implies there is considerable uncertainty with regard to the practical use of the results. Fortunately, the mixed POT-BM approach can provide improved precision with a small confidence interval in the return level estimation. Short return periods less than 10 years can provide satisfactory estimation results for the return level estimates of extreme API events. For moderate return periods between 10 to 50 years, the precision of the estimates decreases as the range of the confidence interval increases. However, information on long-term return periods over than 50 years is not recommended for use as a reference for air pollution management planning and decisions in Klang. Overall, this study concluded that the mixed POT-BM approach is a satisfactory alternative solution for determining extreme data points for the statistical modeling of extreme events, such as unhealthy air pollution.

## 5. Conclusions

This article proposes the use of a novel alternative approach for determining extreme event data called the mixed POT-BM method for extreme-value modeling. A case study was conducted using the API data from Klang, Malaysia, for the period from 1 January 1997 to 31 August 2020. The extreme events of interest corresponded to unhealthy air pollution events with a threshold *u* > 100. Since the threshold *u* > 100 was used as the basis for determining the POT blocks, this approach led to the problem of dependency behaviors among the selected extreme data points, which implied a bias in the statistical modeling process. Thus, to overcome the problem of dependency behaviors present in adjacent POT blocks, a declustering technique was employed. The declustering technique was used by filtering consecutive dependent extreme data points with 240 h minimum separation from one another. It was found that the application of the declustering technique in POT blocks was able to solve the problem of dependency behaviors for selected extreme data. In addition, the BM concept was integrated to determine the maximum data points in each POT block. The extreme data points determined by the mixed POT-BM approach satisfied the independent properties of independent extreme events, with satisfactory fitted model precision results. The estimated parameters for the GEV approximation based on the POT-BM approach were found to produce a considerably lower standard error compared to the original GEV modeling with the BM approach. Apart from that, the return level plot derived from GEV model with the POT-BM approach was also found to produce a good result. The return level estimation based on short return periods of time (5 or 10 years) was found to be more accurate compared to other extreme-value models. Overall, this study concludes that the results obtained by the POT-BM method can provide an improved balance in the tradeoff between bias and variance in extreme-value modeling. Thus, the POT-BM approach is a satisfactory practical application alternative for extreme-value event analysis and modeling.

## Figures and Tables

**Table 1:** Air quality statuses corresponding to API values.

Pollution Index	Status	Health Effect
0–50	Good	Low pollution with no ill effects on health
51–100	Moderate	Moderate pollution that poses no ill effects on health
101–200	Unhealthy	Worsens the health conditions of high-risk individuals with heart and lung complications
201–300	Very unhealthy	Worsens the health conditions and reduces the tolerance to physical exercise of individuals with heart and lung complications; affects public health
>300	Hazardous	Hazardous to high-risk individuals and public health in general

**Table 2:** Descriptive statistics of API data in the Klang area.

Location	Mean	Standard Deviation	Min. Value	Max. Value	Skewness	Kurtosis
Klang	55.222	20.970	0	543	4.537	65.133

**Table 3:** Results of parameter estimation for each fitted extreme-value model.

Model	Parameter Estimated	Standard Error
GEV based on BM	Location = 143.805	13.293
	Scale = 46.404	17.071
	Shape = 0.415	0.127
GPD based on POT	Shape = 0.2933	0.017
	Scale = 23.7401	0.511
GEV approximation based on POT-BM	Location = 110.863	1.234
	Scale = 12.911	1.376
	Shape = 0.788	0.118

## Data Availability

Due to confidentiality agreements, supporting data can only be made available to bona fide researchers subject to a non-disclosure agreement. Details of the data and how to request access are available from the Department of Environment Malaysia, https://www.doe.gov.my/portalv1/en/at (accessed on 4 March 2021).
